# Distinct Top-down and Bottom-up Brain Connectivity During Visual Perception and Imagery

**DOI:** 10.1038/s41598-017-05888-8

**Published:** 2017-07-18

**Authors:** N. Dijkstra, P. Zeidman, S. Ondobaka, M. A. J. van Gerven, K. Friston

**Affiliations:** 1 Radboud University, Donders Institute for Brain, Cognition and Behaviour, 6525 EN Nijmegen, The Netherlands; 2 The Wellcome Trust Centre for Neuroimaging, UCL, 12 Queen Square, London, UK

## Abstract

Research suggests that perception and imagination engage neuronal representations in the same visual areas. However, the underlying mechanisms that differentiate sensory perception from imagination remain unclear. Here, we examine the directed coupling (effective connectivity) between fronto-parietal and visual areas during perception and imagery. We found an increase in bottom-up coupling during perception relative to baseline and an increase in top-down coupling during both perception and imagery, with a much stronger increase during imagery. Modulation of the coupling from frontal to early visual areas was common to both perception and imagery. Furthermore, we show that the experienced vividness during imagery was selectively associated with increases in top-down connectivity to early visual cortex. These results highlight the importance of top-down processing in internally as well as externally driven visual experience.

## Introduction

Visual experience can be caused by external events in the outside world, like the appearance of an object, or by internal signals generating visual images in our mind’s eye. Localisation of the neural structures that represent the content of visual imagery is an important step in the process of understanding the underlying mechanisms that generate visual images^1^. In 1980, Kosslyn proposed that imagery uses the same ‘visual buffer’ as perception to represent visual content. In line with this idea, neuroimaging has shown that visual areas have similar neural representations of imagined and perceived objects, with higher overlap in late visual areas^2, 3^. The overlap in early visual areas depends on the exact imagery task^4, 5^ and the experienced vividness of the imagery^6, 7^.

Developing a detailed understanding of the mechanisms by which our brains generate visual experience calls for the elucidation of dynamic top-down and bottom-up connectivity within and between the neural structures involved^8, 9^. Whereas during perception, activation of visual representations is ultimately caused by bottom-up influences from the retina, these exogenous influences are absent during visual imagery. How visual areas are activated in the absence of stimulus bound, bottom-up input, remains an open question. Recent work using measures of effective (directed) connectivity during imagery suggests that top-down projections from fronto-parietal areas to visual areas are involved in visual imagery^10, 11^.

There is a large body of research showing that top-down influences also play an important role in perception^12–14^. The predictive coding account of perception proposes that visual experience is a product of the reciprocal exchange of bottom-up and top-down influences throughout the neuronal hierarchy^15, 16^. From this perspective, the question arises to what extent recurrent exchange differs during perception and imagery. Here, we investigated this aspect of distributed neuronal processing by examining how effective connectivity changes during these two forms of visual experience.

We hypothesized distinct context sensitive patterns of top-down and bottom-up influences during imagery compared to perception. We used dynamic causal modelling (DCM) to characterise the effective connectivity that best explains the BOLD (Blood Oxygen-Level Dependent) response during visual perception and imagery. Based on hierarchical predictive coding, we hypothesized an increase in bottom-up coupling, relative to baseline, during perception but not imagination and an increase in top-down coupling during visual experience; i.e., both perception and imagery.

## Materials and Methods

### Subjects

Twenty-nine healthy adult volunteers with normal or corrected to normal vision gave written informed consent and participated in the experiment. An initial analysis of these data is already published in Dijkstra, Bosch, and van Gerven^7^. Three participants were excluded; two due to insufficient data caused by scanner problems and one due to not completing the task. Twenty-six participants (mean age = 24.31, *SD* = 3.05, 18 female) were included in the reported analyses. The study was approved by and in accordance with the guidelines of the local ethics committee (CMO Arnhem-Nijmegen).

### Procedure and experimental design

**Figure 1:**
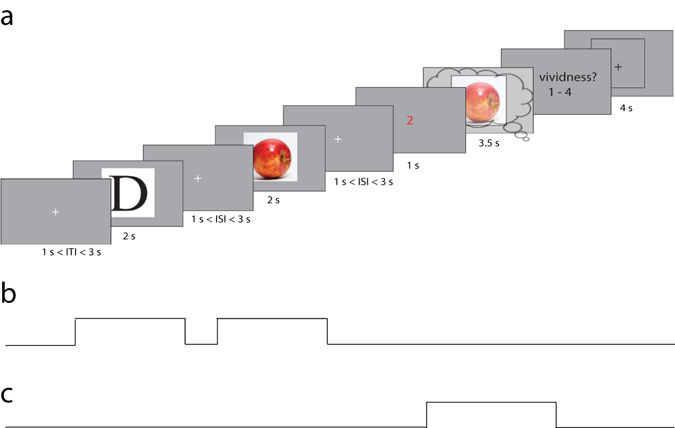
Experimental paradigm. (**a**) Participants were shown two objects for 2 seconds each with a random inter stimulus interval (ISI) lasting between 1 and 3 seconds during which a fixation cross was shown. Next, another fixation cross was shown for 1–3 seconds after which a red cue was presented indicating which of the two objects the participant had to imagine. Subsequently a frame was presented for 3.5 seconds on which the participant had to imagine the cued stimulus. After this they had to rate their experienced imagery vividness on a scale from 1 (not vivid at all) to 4 (very vivid). Each trial was followed by a 4-second baseline period in which there was no perception and no imagery. The apple image can be found at https://commons.wikimedia.org/wiki/File:Red_Apple.jpg, it falls under the CC Attribution 2.0 license (https://creativecommons.org/licenses/by/2.0/) and functions as a placeholder for the original stimulus which cannot be shown due to copyright limitations. (**b**) Boxcar regressor for *perception* used as driving and modulatory input for the DCM. This regressor was on for 2 seconds during the first stimulus presentation, off during the inter-stimulus-interval, and then on again for 2 seconds during the second stimulus presentation. (**c**) Boxcar regressor for *imagery*, this regressor was on for 3.5 seconds during the presentation of the imagery frame.

The experimental paradigm is depicted in Fig. 1a. We adapted a retro-cue working memory paradigm^17^. In each trial, participants were shown two successive images, followed by a cue indicating which of the two they should subsequently imagine. The stimulus set consisted of six images obtained from the World Wide Web: two faces (Barack Obama and Emma Watson), two letters (‘D’ and ‘I’) and two kinds of fruit (banana and apple). During imagery, a frame was presented within which subjects were asked to imagine the cued stimulus as vividly as possible, while maintaining fixation on a cross presented in the centre of the screen. It has been shown that neural activity during visual imagery is influenced by the experienced vividness of visual imagery^2, 6, 18^. Therefore, we asked participants to indicate their experienced vividness of visual imagery on each trial on a scale from one to four, where one was low vividness and four was high vividness. Previous research has shown that such a subjective imagery rating shows high test-retest reliability and correlates with objective measures of imagery vividness^18, 19^.

The experiment comprised nine blocks, each consisting of twenty trials. Hence, each stimulus was perceived 60 times and imagined 30 times over the course of the whole experiment, resulting in a total scanning time of approximately 1.5 hours per participant.

### General linear model

The fMRI acquisition and pre-processing details are described in the Supplementary Material. All analyses were performed using SPM12. To identify our regions of interest, prior to the connectivity analyses, we inverted a general linear model (GLM), with data and regressors concatenated over runs. As our focus was on establishing domain general mechanisms of imagery and perception, we collapsed over stimulus categories, thereby simplifying the subsequent DCM analysis. Our regressors of interest modelled the perception events, the imagery events, and the parametric modulation of imagery vividness. Inspection of the design orthogonality revealed an absolute cosine angle of 0.13 between the perception and imagery regressor, indicating that the experimental design enabled the imagery response to be disentangled from the perception response. Analysis of behavioural data (vividness ratings during scanning) demonstrated an effect of stimulus category on vividness, with letters being experienced as more vivid than fruit and fruit as more vivid than faces^7^. This indicated that any effect of vividness could be explained by stimulus category. To control for contributions of stimulus category, we regressed out the category effect by mean-centring the vividness scores per category. Finally, the visual cues, the presentation of the vividness instruction screen and the button presses were modelled with separate regressors, along with the six movement (nuisance) regressors.

### Selection of regions of interest

We selected regions of interest (ROI) for our connectivity model based on the results from our GLM analysis and prior knowledge from the literature^3, 20, 21^. To allow for variation between participants in the exact location of the effect, we selected the ROIs per participant as an 8 mm sphere centred on the subject-specific maximum within a 16 mm sphere centred on the group maximum, in space. This approach ensures that the subject- specific ROIs were close to the average group activity, while allowing for slight variation in functional anatomy between participants.

In each subject, time series were extracted from every voxel in each of the ROIs. The data were subsequently adjusted based on an F-contrast that retained the experimental effects of interest (i.e., perception, imagery, vividness, cue, button press, instruction text) and regressed out task-unrelated variance caused by sources that were not of interest, for example, head movement. The first principal component (eigenvariate) of each region’s adjusted data was then used as the time series for subsequent DCM analysis.

### Dynamic causal modelling

In order to quantify the effects of perception, imagery and vividness on effective connectivity between the ROIs, we used dynamic causal modelling (DCM)^22^. DCM uses the following bilinear state equation to infer effective connectivity parameters:

1 $$\dot{\,z}=\,(A+\,\sum _{j=1}^{M}{u}_{j}{B}^{j})\,z+Cu$$

where the dot notation denotes the time derivative. The variable *z* describes neuronal activity resulting from the interplay of three different influences. First, the *A* matrix represents endogenous or fixed connectivity during baseline, in the absence of external stimulation. Second, the elements in *B* ^*j*^ represent the changes in connectivity due to experimental influences *u*_*j*_. Finally, the matrix *C* denotes the direct influence of each experimental input *u*_*j*_. The neuronal model is coupled to a biophysically plausible model of neurovascular coupling and the BOLD response, which together generate the predicted BOLD time series. The slice timing model within DCM was set to its default value of 0.5 TR^23^. The model is then inverted to find the neural and haemodynamic parameters which offer the best trade-off between model fit and complexity (i.e. maximises the negative free energy, which is an approximation to the log model evidence log *p*(*y|m*))^24^.

A parameter-estimate A_i,j_ of a connection from area i to area j, reflects the rate of change (in Hz) in the neuronal activity of area j that is caused by a change in the firing rate of area i. In this context, a positive parameter refers to an increase in the firing rate of area j caused by area i, which can be interpreted as an excitatory influence. A negative parameter refers to a decrease in the firing rate of area j caused by area i, which can be interpreted as an inhibitory influence. Note that this does not relate to specific excitatory or inhibitory neurons or their axonal projections; rather it is the net influence of one brain region on another. Similarly, a parameter-estimate B_i,j,k_ of an experimental influence k on the connection from area i to j, reflects the increase (in Hz) in the coupling from area i to area j caused by the experimental manipulation k.

### Connectivity parameter estimation

Typically, in DCM, a few models are specified, each of which represents a hypothesis about the connectivity architecture of the system being studied. These models differ in the presence or absence of an influence of experimental manipulations on certain connections. However, in the current context we expected almost all connections to be influenced by perception and imagery, but that the *strength* of influence would be different. In other words, our experimental questions were about the relative strength of coupling under different experimental manipulations, instead of the presence or absence of context sensitive changes in coupling. In other words, our aim was to test quantitative hypotheses about model parameters rather than qualitative aspects of model structure. To test hypotheses about changes in connectivity at the group level, we used hierarchical Bayesian modelling and averaging. This entails evaluating a large number of connectivity models and taking a weighted average of the connectivity parameters, weighted by the evidence of each model^25^. This approach, called Bayesian Model Averaging (BMA), accommodates uncertainty about the underlying model structure on the parameter estimates.

Estimating the parameters of all plausible DCMs can be a time consuming process and novel ways have been developed to make BMA more computationally efficient. Instead of estimating every possible model separately, it is possible to only estimate a full or parent model, containing all the parameters of interest and use the posterior estimates of this full or parent model to derive the posterior estimates (and model evidence) of reduced models, in which one or more parameters are systematically removed. This is called Bayesian model reduction (BMR) and gives similar results to the standard approach, but is computationally much more efficient^25^.

The BMA scheme described above could be used with classical inference methods such as t-tests to evaluate whether a parameter is non-zero at the group level. However, this ignores uncertainty at the within subject level (and assumes that every subject contributes an equally good estimate of the parameters). To circumvent this issue, we used a parametric empirical Bayesian (PEB) approach with DCM^26, 27^. In short, the PEB scheme uses BMR to invert a hierarchical (Bayesian) model of between subject effects on within-subject parameters. This involves specifying a general linear model of between-subject effects. Here, we simply tested for nontrivial group means of condition-specific changes in connectivity (i.e., input specific *B* ^*j*^ parameters above). Further details on how we used PEB in the current analysis can be found in the Supplementary Material.

### Modelling driving inputs and changes in connectivity

In DCM, the activity in the network is driven by direct experimental inputs *u*_*j*_, here modelled as boxcar regressors (Fig. 1b,c). We assume that during perception, sensory input enters the cortical network via the early occipital cortex (OCC). We expressed this in the DCM by modelling the perception regressor (Fig. 1b) as driving the dynamics via region OCC. Imagery, in contrast, is assumed to be internally driven, via top-down coupling from higher areas. However, because we did not want to bias the model towards a top-down account of imagery (and because we did not have sufficiently strong prior beliefs about whether imagery should arise in parietal or frontal areas), we used BMR over all possible driving locations for imagery, prior to estimating condition-specific changes in connectivity (see *Driving input during imagery*).

The main aim of this study was to investigate changes in top-down and bottom-up connectivity between visual perception and imagery. Research on connectivity during visual imagery has been surprisingly scarce. Functional connectivity studies of working memory maintenance, which is assumed to show a substantial overlap with the neural mechanisms of visual imagery^6, 28^, suggests strong connectivity among all our ROIs^29^. Thus, we have no *a priori* reasons to constrain our model. Therefore, we modelled both imagery and perception as potentially influencing all connections. We also had no reason to constrain which connections could be influenced by imagery vividness: more vivid imagery could be caused by an increase in top-down processing from either parietal or frontal areas to early or late visual areas, or by a decrease in bottom-up processing in the same pathways. Furthermore, since most effects of vividness have been found in early visual areas^2, 6, 30^, this could also be due to an increase in excitability within this area as a result of decreased self-inhibition^31^. Therefore, we modelled vividness on all connections and on the self-connection of OCC. We used the analysis scheme described above with PEB and BMR to estimate differential (group average) changes in directed connectivity within the DCM.

### Data availability

The data that support the findings of this study are available from the corresponding author upon reasonable request.

## Results

### Imagery vividness ratings

The mean vividness rating over trials, averaged over participants, was 2.97 (SD = 0.52). All participants showed variation in their vividness ratings over the course of the experiment (mean SD = 0.79, SD = 0.17). On average, 8.79% of trials were rated as vividness = 1, 20.85% as vividness = 2, 35.10% as vividness = 3 and 35.28% as vividness = 4.

### Activated brain areas during perception and imagery

**Figure 2:**
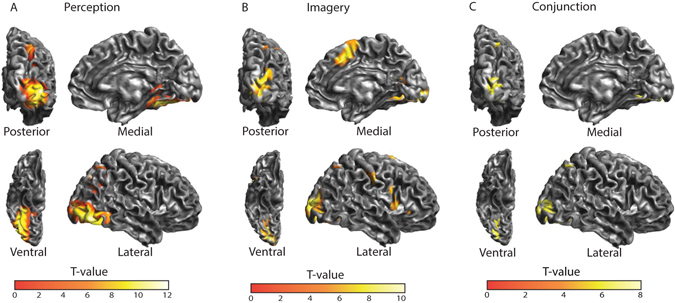
Activated brain areas. Activations shown are significant on the group level (p < 0.05; FWE corrected) with a cluster forming threshold of 50 voxels. (**A**) Perception versus baseline. (**B**) Imagery versus baseline. (**C**) Conjunction between perception and imagery.

The standard SPM (GLM whole brain) analysis showed that both perception and imagery activated early visual, ventral stream and parietal areas (Fig. 2). Furthermore, imagery was associated with a large increase in activity in inferior frontal gyrus (IFG) and supplementary motor area (SMA). SMA responses were possibly due to motor preparation for the vividness rating; therefore, we did not include this area in further analysis. We focused on the right hemisphere to reduce model complexity and because it has been shown that imagery is associated with stronger activation of the right hemisphere^32^.

Based on these regionally specific effects, we defined four ROIs: early visual cortex (OCC), late visual cortex/fusiform gyrus (FG), intraparietal sulcus (IPS) and inferior frontal gyrus (IFG). OCC, FG and IPS regions were based on the conjunction between the main effect of imagery and the main effect of perception and IFG was selected based on the main effect of imagery (Table 1).

**Table 1:** ROIs used in the connectivity analysis.

Region of interest	MNI coordinates			T-value relevant effect
	x	y	z	
Early visual cortex (OCC)	28	−94	4	7.22
Fusiform gyrus (FG)	33	−60	−13	6.43
Intraparietal sulcus (IPS)	33	−59	53	6.02
Inferior frontal gyrus (IFG)	46	17	2	6.14

### Driving input during imagery

**Figure 3:**
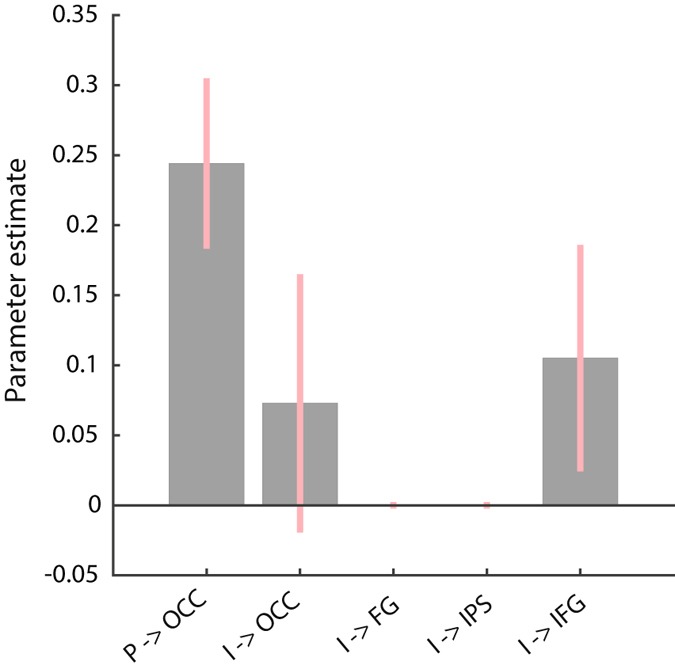
Bayesian model reduction for the driving input of imagery. P: perception, I: imagery. The mean of the group level parameter estimates after BMR are shown. The estimated parameters have a multivariate normal distribution, specified by their means (gray bars) and covariance. We transformed the estimated variance of the parameters to 95% confidence intervals, which are displayed as the pink bars. The results show that the driving input during imagery can be best modelled as targeting both IFG and OCC.

To identify where imagery exerts the driving input (Fig. 1c), we constructed a group-level (PEB) model on the driving input parameters (C-matrix). We used BMR, to prune any driving effects not contributing to the model evidence. The largest and most reliable effect was perception driving OCC activity (Fig. 3). With respect to imagery, the model with the greatest evidence suggested that imagery drove activity in IFG – and there was no evidence for driving effects on FG or IPS. There was also some evidence for direct imagery input in OCC, but the 95% confidence interval of this parameter included zero, so we cannot be confident about this effect. This result could relate to the fact that, in our set-up, the imagery period started with a visual cue that initiated the imagery response. Based on these results, and because we did not want to bias the model towards a top-down account of imagery *a priori*, we specified the driving input during perception on OCC and during imagery on both IFG and OCC, for the subsequent DCM analysis of quantitative changes in effective connectivity.

### Influence of perception and imagery on connectivity

**Figure 4:**
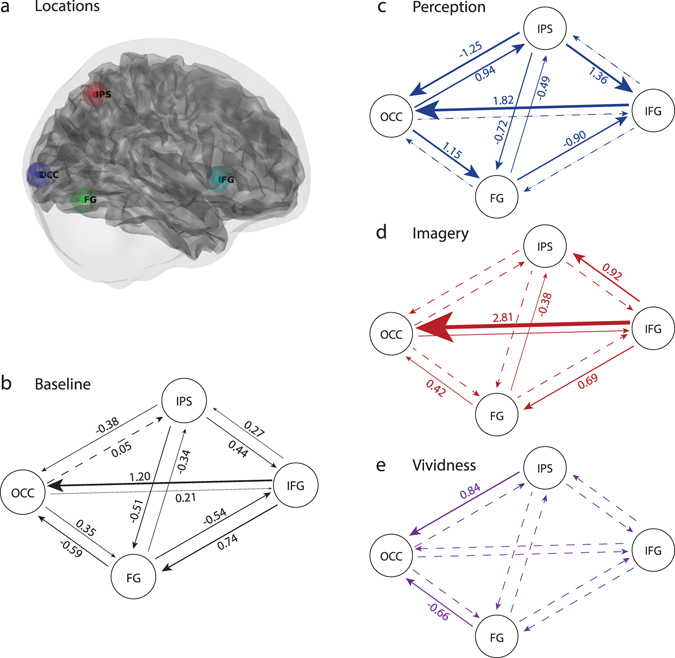
Influences on effective connectivity. Connections with solid lines had a posterior probability (Pp) of at least 0.95. The numbers indicate the strength of directed coupling (in Hz), with a minus sign indicating inhibitory influences. The width of the arrows is proportional to the strength of coupling. (**a**) Locations of the different ROIs for one subject. (**b**) Parameters of the A matrix which reflects the connectivity during baseline, i.e. in the absence of experimental influences. (**c**) The effect of perception. (**d**) The effect of imagery. (**e**) The effect of vividness.

We next performed a group-level analysis on the baseline connectivity and its modulation by perception, imagery and imagery vividness (modelled as a parametric regressor). As before, we estimated a group level PEB model and pruned any connections not contributing to the model evidence. Significance in this context is defined as a posterior probability (Pp) of at least 0.95, based on the (family) comparison of models in which the parameter was switched on, versus models in which it was switched off. The results from the BMR on all connections are shown in Fig. 4. During baseline, in the absence of experimental input, all connections were significant except the OCC to IPS connection (Fig. 4b).

The effects of perception and imagery were modelled as changes in connectivity relative to baseline coupling. Perception had an influence on most connections throughout the network; one clear example is the coupling from OCC to IPS (Fig. 4c). During baseline, there was only a small influence of OCC on IPS (0.05 Hz change in firing rate) but during perception, there was a very strong excitatory effect (0.94 Hz). The strongest effect of imagery was an increase in top-down excitatory coupling from IFG to OCC, more than tripling with respect to baseline (Fig. 4d). Note that this connection was also significantly increased during perception. Finally, vividness was only associated with modulation of top-down coupling to OCC, switching the influence of IPS on OCC from inhibition to excitation, and increasing the inhibitory influence of FG on OCC (Fig. 4e).

### Top-down versus bottom-up coupling

**Figure 5:**
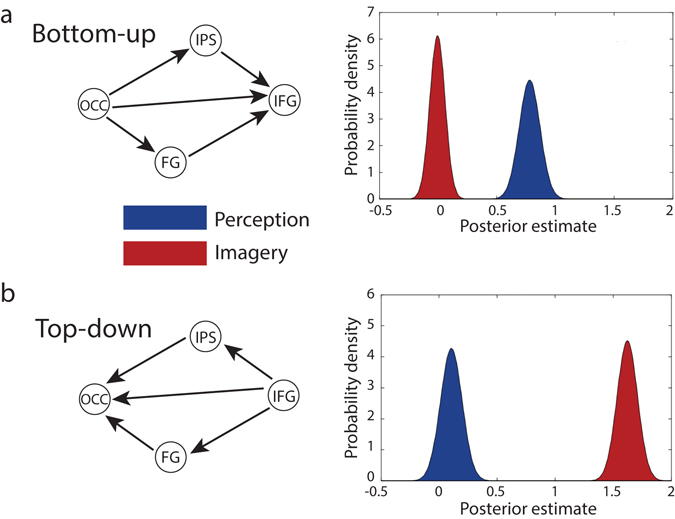
Top-down versus bottom-up coupling. Posterior densities of (contrasts or mixtures of) parameter estimates of the effect of perception and imagery pooled over bottom-up and top-down connections. (**a**) Coupling during perception and imagery for bottom-up connections. (**b**) Coupling for top-down connections.

The main objective of this study was to characterise differences between perception and imagery in terms of their effect on the strength of top-down and bottom-up coupling. To quantify this differential effect, we pooled over bottom-up and top-down connections. We defined bottom-up (forward) coupling as outgoing connections from OCC and incoming connections to IFG, whereas top-down (backward) coupling was defined as incoming connections to OCC and outgoing connections from IFG. The posterior distribution over this contrast (see Supplementary Material) comparing the strength of bottom-up (Fig. 5a) and top-down (Fig. 5b) coupling during perception and imagery was then evaluated.

We found stronger bottom-up coupling during perception as compared to imagery (Posterior probability (Pp) = 1.0). Furthermore, whilst bottom-up coupling was clearly stronger during perception than baseline (Pp = 1.0), this was not the case during imagery (Pp = 0.48). In contrast, during both perception (Pp = 0.87) and imagery (Pp = 1.0) the top-down coupling was stronger than baseline. Note that the net top-down coupling during perception was close to zero because it was a mixture of excitatory and inhibitory connections (Fig. 4c). Furthermore, top-down coupling was much stronger during imagery than during perception (Pp = 1.0).

## Discussion

The aim of the current study was to characterise bottom-up and top-down coupling during perception and imagery by examining changes in effective connectivity. We found that during perception there was an increase in both bottom-up and top-down coupling relative to baseline. In contrast, during imagery, there was only an increase in top-down coupling – and this increase was much stronger than during perception. These findings illustrate that distinct dynamic top-down and bottom-up mechanisms underlie visual experience during perception and imagery. We show that during perception, an interplay between top-down and bottom-up influences is responsible for visual experience. In contrast, during imagery, where bottom-up drive is absent, there is an increase of top-down coupling that accompanies the visual experience.

Examination of the connectivity parameters shows that the observed increase in top-down coupling during perception was a mixture of inhibitory and excitatory influences. The most prominent excitatory connection was apparent from inferior frontal gyrus (IFG) to early visual areas (OCC); this was also the connection that showed the strongest effect during visual imagery. Coupling from IFG to OCC has been proposed as a common top-down mechanism for different cognitive processes during visual working memory^33^. Top-down connections from the IFG are important for selective attention during encoding^34, 35^ as well as for the maintenance of visual information during the delay period^36, 37^. Our results support the idea that this coupling reflects a general top-down mechanism responsible for enhancing the relevant visual representations in early visual areas: both in the presence as well as in the absence of bottom-up sensory input, subserving perception and imagery, respectively. In this context, the increase in strength during imagery could reflect the increase in general attentional load associated with (internally) generating a visual experience in the absence of sensory input.

The largest inhibitory top-down modulation during perception was an increase in the inhibitory connection from IPS to OCC. We also observed a strong increase in the excitatory bottom-up connection from OCC to IPS. Findings of increased inhibitory top-down influence together with an increase in excitatory bottom-up influence supports a predictive coding view of perception that proposes reciprocal top-down and bottom-up influences during perception^38, 39^. Within this framework, perceptual synthesis arises from an interplay between inhibitory top-down predictions and excitatory bottom-up prediction errors, iteratively leading to an accurate representation of the outside world^40, 41^.

Within hierarchical predictive coding, imagination (and related phenomena such as visual dream content) is thought to reflect the generation of sensory predictions in the absence of precise sensory constraints. In biological implementations of predictive coding, the precision of sensory constraints (i.e., prediction error) is encoded by the gain of bottom-up prediction errors. This formulation of perception and imagination fits very comfortably with our results. Our findings are consistent with an increase in the precision of ascending prediction errors during perception – that induce descending predictions – but not imagination; where sensory precision is reflected in the strength of bottom-up effective connectivity at low levels of the visual hierarchy.

Surprisingly, we did not observe increased top-down coupling from IPS during imagery. This is in contrast with the results from other studies on effective connectivity during visual imagery^10, 11^. However, we did find a significant modulation of this connection by the experienced vividness of the imagery, with more vivid imagery being associated with more excitatory influences from IPS to OCC. During baseline and during perception, coupling from IPS to early visual areas was inhibitory. This means that an increase in imagery vividness was associated with a relative decrease in inhibitory top-down influences (i.e., disinhibition). This might reflect an imagery specific mechanism responsible for preserving early visual cortical activation. The extent to which this succeeds is then reflected in the experienced imagery vividness. On average, this effect may be too subtle to lead to a main effect of imagery.

Finally, we found that imagery vividness was specifically related to the strength of top-down coupling to the early visual cortex. More vivid imagery was associated with a decrease in inhibition from IPS and an increase in inhibition from FG. The fact that only connections to early visual cortex were modulated by vividness is in line with the findings of previous studies that the vividness of visual imagery is specifically associated with early visual cortex activity^2, 6, 18^. Here, we extend previous findings by revealing a possible mechanism for this observation. Decreased inhibitory influences of IPS might allow activation in early visual cortex engendered by top-down signals from IFG to be better preserved. The increase in inhibitory influence from FG might in turn reflect top-down sharpening of OCC activity mediated by the selection of (or biased competition among) category representations in FG^42, 43^.

It could be argued that the assumption we made to model sensory information as entering the network during perception via early visual cortex is too simplistic. Afferent neurons from the retina also project directly to the superior colliculus^44–47^, which in turn projects to large parts of the cortex to initiate and guide orienting movements^48–50^. Therefore, it is possible that part of the sensory input entered the network via other cortical regions. This simplifying assumption should be kept in mind when interpreting our findings and future research should explore the effects of changing the locus of visual input during perception.

In conclusion, our results reveal both differences and similarities in the neural mechanisms underlying perception and imagery. Modulation of bottom-up connections was specific for perception, while top-down coupling was increased during both perception and imagery, albeit much more during imagery. Coupling from frontal to early visual areas seems to represent a common mechanism during perception and imagery, whereas the role of top-down connections from parietal cortex is less clear: the inhibitory influence increases during perception and decreases with more vivid imagery.

## Electronic supplementary material

Supplementary material
