# Supplementary material for: Distinct Top-down and Bottom-up Brain Connectivity During Visual Perception and Imagery
Source: Sci Rep. 2017 Jul 18;7:5677. doi: 10.1038/s41598-017-05888-8 (PMC5516016; doi:10.1038/s41598-017-05888-8)
Supplement: Supplementary file 1 — Supplementary material [file 41598_2017_5888_MOESM1_ESM.pdf]

Supplementary Material

Title: Distinct Top-down and Bottom-up Brain Connectivity During Visual Perception and Imagery

Authors: N. Dijkstra*[1], P. Zeidman[2], S. Ondobaka[2], M.A.J. van Gerven[1]., & K. Friston[2]

Affiliations:

1. Radboud University, Donders Institute for Brain, Cognition and Behaviour, 6525 EN, Nijmegen, the Netherlands.

2. The Wellcome Trust Centre for Neuroimaging, UCL, 12 Queen Square, London, UK.

* Contact information: n.dijkstra@donders.ru.nl; Montessorilaan 3, 6525 HR Nijmegen; tel. 003124-3655933

Methods

FMRI acquisition. Each block was acquired in a separate fMRI run, leading to 9 runs in total. In between runs the participant had a break and indicated – by means of a button press – when they were ready for the experiment to continue. FMRI data were required on a Siemens (Erlangen, Germany) 3T Prisma scanner with a Multiband factor 4 sequence (TR: 1.5 s; voxel size: 2 × 2 × 2 mm; TE: 39.6 ms) and a 32-channel head coil. For all participants the field of view was tilted -25° from the transverse plane, using the Siemens AutoAlign Head software, resulting in the same tilt relative to the individual participant's head position. T1-weighted structural images (MPRAGE; voxel size: 1 × 1 × 1 mm; TR: 2.3 s) were also acquired for each participant.

FMRI data pre-processing. Data were pre-processed using SPM8. Functional imaging data were motion corrected and co-registered to the T1 structural scan. Next, the data were normalised to MNI space and smoothed with an 8 mm smoothing kernel. During subsequent GLM analysis, we used a high-pass filter of 128 s to remove slow signal drift.

Bayesian contrasts. To be able to draw inferences about mixtures or contrasts of model parameters, i.e. whether one parameter set was larger than another one, we computed Bayesian contrasts. For example, to assess if the first parameter in the model is larger (more positive) than the third parameter, the contrast, c, is a vector:

$$(1)\ c = [1, 0, -1]'$$

The posterior mean of the contrast is calculated by multiplying c by a vector E, which contains the estimated mean of each parameter:

$$(2)\ \mu = c^{\prime}\ E$$

The posterior variance of the contrast is calculated similarly, using estimated covariance matrix V:

$$(3)\ \sigma^2 = c^{\prime}\ Vc$$

To report the results (Figure 6), we plotted the probability density function $N(\mu, \sigma^2)$, which is the posterior probability of a given (Bayesian) contrast. Note that all parameters and contrasts thereof are normally distributed as DCM uses a variational scheme under Gaussian assumptions about posterior is; namely, the Laplace assumption.

Parametric empirical Bayes: we applied parametric empirical Bayes (PEB) at two different points in the analysis process. First, when estimating the full DCM model for each subject, we re-initialised model fitting multiple times using the group-level (PEB) parameters as empirical priors. This has the effect of ‘pulling’ individual subjects’ parameter estimates to the global maximum; thereby leading local maxima. Second, after the full model was estimated for each participant, we used BMR combined with a greedy search to compare the full or parent DCM against reduced DCMs, where combinations of parameters were switched off. This greedy search procedure iteratively prunes parameters that do not contribute to the model evidence from the model. Finally, we averaged the parameters over reduced DCMs (weighted by their evidence) to obtain a set of Bayesian model averages (BMA), representing the strength of each connection and the effects of experimental manipulations. The ensuing (BMA) posterior densities for each subject were then used to estimate the posterior densities over group means using the second instance of PEB. We evaluated the evidence for each connectivity parameter (at the group level) by comparing the evidence for PEB models in which the parameter was switched on, versus models in which it was switched off. This provides the posterior probability that each connection (or change in connection) is necessary to explain the group data.
